# Large optical nonlinearity enabled by coupled metallic quantum wells

**DOI:** 10.1038/s41377-019-0123-4

**Published:** 2019-01-23

**Authors:** Haoliang Qian, Shilong Li, Ching-Fu Chen, Su-Wen Hsu, Steven Edward Bopp, Qian Ma, Andrea R. Tao, Zhaowei Liu

**Affiliations:** 1 Department of Electrical and Computer Engineering, University of California, San Diego, 9500 Gilman Drive, La Jolla, CA 92093 USA; 2 Department of NanoEngineering, University of California, San Diego, 9500 Gilman Drive, La Jolla, CA 92093 USA; 3 Materials Science and Engineering, University of California, San Diego, 9500 Gilman Drive, La Jolla, CA 92093 USA; 4 Center for Memory and Recording Research, University of California, San Diego, 9500 Gilman Drive, La Jolla, CA 92093 USA

**Keywords:** Nonlinear optics, Nanophotonics and plasmonics

## Abstract

New materials that exhibit strong second-order optical nonlinearities at a desired operational frequency are of paramount importance for nonlinear optics. Giant second-order susceptibility *χ*^(2)^ has been obtained in semiconductor quantum wells (QWs). Unfortunately, the limited confining potential in semiconductor QWs causes formidable challenges in scaling such a scheme to the visible/near-infrared (NIR) frequencies for more vital nonlinear-optic applications. Here, we introduce a metal/dielectric heterostructured platform, i.e., TiN/Al_2_O_3_ epitaxial multilayers, to overcome that limitation. This platform has an extremely high *χ*^(2)^ of approximately 1500 pm/V at NIR frequencies. By combining the aforementioned heterostructure with the large electric field enhancement afforded by a nanostructured metasurface, the power efficiency of second harmonic generation (SHG) achieved 10^−4^ at an incident pulse intensity of 10 GW/cm^2^, which is an improvement of several orders of magnitude compared to that of previous demonstrations from nonlinear surfaces at similar frequencies. The proposed quantum-engineered heterostructures enable efficient wave mixing at visible/NIR frequencies into ultracompact nonlinear optical devices.

Large second-order optical nonlinearity in the infrared frequency range has been achieved by leveraging electronic intersubband transitions in semiconductor quantum well (QW) heterostructures^1–5^. However, it is problematic to extend this mechanism into the visible/near-infrared (NIR) frequencies, where the majority of optical nonlinearities play crucial roles in optoelectronics and photonics^6–8^. Recent advances in ultrathin gold films introduce a possible solution for the creation of giant nonlinearities in a higher energy range of the spectrum due to the large depth of these QWs^9^. However, restricted by the accessible growth technologies, these ultrathin gold films cannot be used for sophisticated coupled QW heterostructures as epitaxial semiconductor wells.

In a different context, transition metal nitrides have recently received increased attention for their use in plasmonics^10,11^. These plasmonic materials have optical responses similar to those of gold while showing vastly enhanced chemical and thermal stabilities^12,13^, rendering them suitable for the study of optical nonlinearity. Among these materials, titanium nitride (TiN) can exist in a range of nonstoichiometric compositions and can be grown epitaxially on various transparent substrates such as sapphire^14,15^. Therefore, epitaxial heterostructures of TiN/Al_2_O_3_ have become excellent candidates for obtaining ultra-large *χ*^(2)^ in the frequency range from visible to NIR spectra.

Here, we report an extremely high *χ*^(2)^ of approximately 1500 pm/V in the NIR frequency range for a visible-frequency second harmonic generation (SHG) due to the electronic intersubband transition of asymmetric coupled metallic QWs (cMQWs) made of TiN/Al_2_O_3_ epitaxial multilayers. This value is more than one order of magnitude higher than that of traditional nonlinear crystals, and it is a few orders of magnitude higher than that of classic plasmonic metal structures^16^. The cMQWs were carefully designed to support three electronic subbands with equal energy spacing (i.e., *E*_*i*_ − *E*_*i*−1_ = *E*_*i*+1_ − *E*_*i*_ = ℏΩ, where ℏ is the reduced Planck constant and Ω is the double transition frequency), such that *χ*^(2)^ near the double transition frequency Ω is^17^

1 $${\hskip -17.5pt}{{\mathrm{\chi }}^{(2)}\left( {\mathrm{\omega }} \right) = \frac{{n_{i - 1} - n_i}}{{\hbar ^2\varepsilon _0}}\frac{{e^3z_{i - 1,i}z_{i,i + 1}z_{i + 1,i - 1}}}{{({\mathrm{\omega }} - {\mathrm{\Omega }} - i{\mathrm{\Gamma }}_{i,i - 1})(2{\mathrm{\omega }} - 2{\mathrm{\Omega }} - i{\mathrm{\Gamma }}_{i + 1,i - 1})}}}$$

where *n*_*i*_, *ε*_0_, and *e* represent the electron density in the *i*th subband, the vacuum permittivity, and the electron charge, respectively. The dipole moment *ez*_*i*,*j*_ and the decay rate Γ_*i*,*j*_ are associated with optically active subbands. Only the lowest of the three electronic subbands *E*_*i*−1_ was below the Fermi level *E*_F_ in order to increase the electron density of (*n*_*i*−1_ − *n*_*i*_) and thus *χ*^(2)^. The product of the dipole moments *e*^3^*z*_*i*−1,*i*_*z*_*i*,*i*+1_*z*_*i*+1,*i*−1_ is large in asymmetric QWs^17^, which also contributes to a large *χ*^(2)^. In addition, a significantly increased *χ*^(2)^ is exhibited in the cMQWs when the incident frequency *ω* is resonant with the double transition frequency Ω. Moreover, the peak *χ*^(2)^ can be tuned to the visible/NIR spectrum due to the extreme depth of the metallic QW. Therefore, due to the large dipole moment and the high carrier concentration within a single QW, and the double resonant transition between equally spaced electronic bound states formed by the coupled QWs, we expect to observe a giant *χ*^(2)^ in the visible/NIR range of the spectrum in these proposed cMQWs.

Based on the above principle, a single cMQW unit consists of two TiN metallic wells with different thicknesses separated by an ultrathin Al_2_O_3_ dielectric barrier, as shown in Fig. 1. Using the recently proposed quantum electrostatic model of quantum sized metals^9^, in order to produce an SHG output at the visible frequency of 460 nm (2Ω), the widths of these wells (1.0 and 2.2 nm) and the barrier (0.5 nm) were chosen to have a double transition frequency Ω at 920 nm. The TiN/Al_2_O_3_ cMQWs were fabricated via an epitaxial growth method using the reactive magnetron sputtering technique (AJA International) (see Supplementary Section S1). Figure 1b shows a transmission electron microscope (TEM) cross-section of one sample consisting of four cMQW units. Here, the units are clearly visible and well-grown, with uniformity over a large area. A dark-field high-resolution TEM (HRTEM) image of one cMQW unit showed atomic level accuracy in the thickness of both the metal and dielectric epitaxial films (Fig. 1b, inset). The use of such a high-quality metal/dielectric epitaxial heterostructure as the material platform of these cMQWs ensures the precise control of intersubband quantum engineering for extremely high optical nonlinearities at visible/NIR frequencies.

**Fig. 1:**
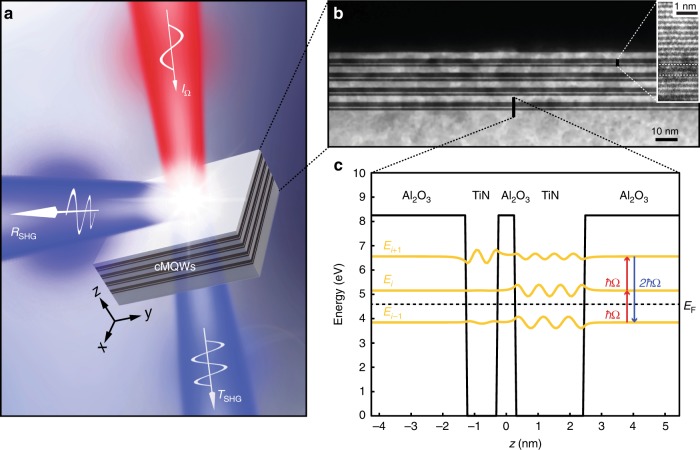
cMQWs for extreme optical nonlinearities and highly efficient visible-frequency SHG. **a** Schematic that illustrates how incident light *I*_Ω_ is converted into the reflected *R*_SHG_ and transmitted *T*_SHG_ SHG waves by cMQWs-based ultrathin film, where phase matching is automatically satisfied. **b** TEM cross-section of an ultrathin film system with 4 units of the cMQWs. The layer on top of the cMQWs is a protective layer used only for TEM cross-section preparation during the focused ion beam (FIB) cutting process. The inset shows a dark-field HRTEM image where atomic-level epitaxial layers are clearly visible. **c** Conduction band diagram of a single cMQW unit. The electron wavefunctions (yellow lines) of three subbands near the Fermi level *E*_F_ (~4.6 eV) are plotted. These subbands are designed to provide a double resonant transition (ℏΩ), which gives rise to the desired SHG (2ℏΩ) at the visible frequency

The equipment and apparatus used in the SHG measurement are schematically depicted in Fig. 2a. We used 100-fs pulse width and 80-MHz repetition rate laser pulses (Mai Tai HP) to excite the SHG. A 50× objective (NA = 0.8, Olympus IX81) was used to focus the incident pulse and collect the reflected wave. The incident power of the pulsed laser was measured by a power meter (Vega), while the excited SHG emission power was collected at both the transmission and reflection sides by a photon counting detector (Horiba PPD). The angular SHG emission distribution was obtained at the Fourier plane with a charge-coupled device (CCD; Andor iXon EMCCD). Due to the polarization selection rule of intersubband transitions in a planar QW structure, only the electric field component *E*_*z*_ of the pump light contributes to the optical responses of the QW (including the SHG). Therefore, an in-plane polarized pump light (i.e., polarization angle *φ* = 90°) is obliquely incident on the sample surface with a fixed incident angle of *θ* = 30° (Fig. 2b, left inset). Here, samples with a single cMQW unit were investigated to verify the origin of SHG. Figure 2b shows a measured emission spectrum reflected from such a sample as excited by a 920-nm (Ω) light pulse with an average power of 3 mW, which corresponds to a peak intensity of 0.48 GW/cm^2^. Note that the power average is taken over one cycle period, which, knowing the beam spot size (5 μm in radius), can be converted to the pulse intensity. We clearly observe that the spectrum features a sharp peak centered at the SHG wavelength of 460 nm (2Ω). It is worth noting that SHG signals from the substrate (sapphire) and the thick TiN film (30 nm) are below the noise floor under the same experimental conditions. The measured dependence of the total (transmitted and reflected) SHG emission intensity on the incident power is shown in the right inset of Fig. 2b, which agrees well with the quadratic prediction^6^.

**Fig. 2:**
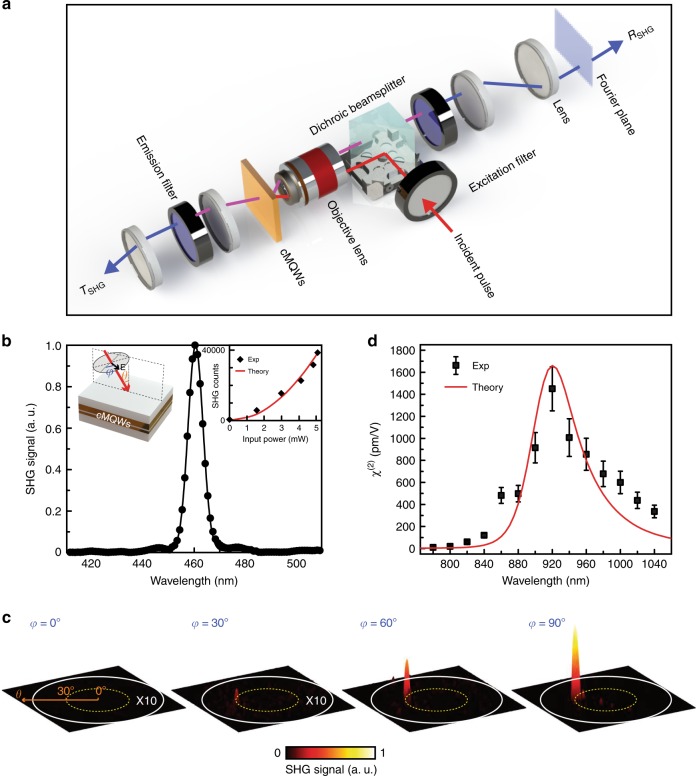
Experimental characterization of visible-frequency SHG enabled by cMQWs. **a** Schematic diagram of the SHG measurement. **b** SHG emission spectrum under a 920-nm light pulse (100-fs pulse width, 80-MHz repetition rate, 5-μm spot radius) excitation with an average power of 3 mW (peak intensity of 0.48 GW/cm^2^). Solid black circles are the experimental result, which is fitted by a Gaussian lineshape function (black line). Left inset shows the experimental configuration where the pump light (thick red arrow) with the electric field **E** (black arrow), polarized at angle *φ* to the out-of-plane (with respect to the incident plane) direction, is obliquely incident on the sample surface with the incident angle *θ*. Right inset shows the total (transmitted and reflected) SHG emission at various incident powers. The experimental results fall on the quadratic theoretical curve. **c** CCD images at the back-aperture plane of the objective (equivalent to the Fourier plane) showing the evolution of emissions at 460 nm as the polarization angle *φ* and thus *E*_*z*_ increases. The dashed yellow circle highlights the collection angle of *θ* = 30°, while the solid white circle indicates the maximum collection angle of the objective. **d** Wavelength dependence of *χ*^(2)^ for a single unit of the cMQWs. The error bars represent the variation in the mean value measured over ten different locations on three identical samples. The presence of a resonant peak for the wavelength-dependent *χ*^(2)^ is evidence of the double resonant transition in the single cMQW unit

In general, phase matching is a critical requirement for an SHG nonlinear optical process. However, because the propagation distance (less than 10 nm for the single cMQW unit) is significantly shorter than the SHG coherence length, the phase-matching condition is automatically satisfied in this cMQW system. Consequently, distinct visible-frequency SHG signals in both the reflection and transmission directions were clearly observed (Fig. 1a, Fig. S1 in Supplementary Section S2). To determine the directionality of the SHG emission, a CCD at the back-aperture plane of the collection objective was used to measure the emission signal (Fig. 2a). Figure 2c shows the back-aperture images at four different incident polarization states from the out-of-plane (with respect to the incident plane, *φ* = 0°) to the in-plane (*φ* = 90^ο^), with a fixed incident angle of *θ* = 30°. The SHG signals exhibit a sharp peak localized at the collection angle of *θ* = 30° in the back-aperture plane. This demonstrates that the SHG signals are directly aligned with the reflection of the incident light (not a photoluminescence process), as illustrated in Fig. 1a. Under the pulse excitation with 0.48-GW/cm^2^ peak intensity, SHG emission has a sin^4^(*φ*) dependence arising from the intersubband transition polarization selection in QW structures^18^, which states that SHG from an intersubband transition is proportional to the square of the out-of-plane polarization intensity *I*_*z*_ which scales as *E*^2^sin^2^(*φ*), where *E* is the incident electric field.

The giant *χ*^(2)^ for visible-frequency SHG enabled by cMQWs relies on the double resonant transition. Figure 2d shows the experimentally obtained *χ*^(2)^ (Supplementary Section S3) compared with the theoretical prediction from Eq. (1)^6,9,19^. This *χ*^(2)^ spectrum has a resonant peak centered at the double transition frequency Ω (920 nm) as expected in Fig. 1c, which indicates the intersubband resonant transition enhancement by the cMQWs. At the NIR frequency, the *χ*^(2)^ is as high as 1500 pm/V, which is more than 20 times higher than that of traditional nonlinear crystals (75 pm/V for LiNbO_3_) and is several orders of magnitude higher than that of typical metal structures (~1 pm/V)^16^. Such a large *χ*^(2)^ at NIR frequency shows that the cMQWs are excellent candidates for ultracompact nonlinear components.

Plasmonically enabled electric field enhancements have recently been used in conjunction with micro- and nanostructures whose characteristic lengths are far below the light wavelength (i.e., should overcome the diffraction limit imposed on a conventional dielectric resonance structure)^20^, leading to ultracompact nonlinear optoelectronic devices^21^. Plasmonic enhancement could be combined with the ultra-large *χ*^(2)^ for surface SHG at visible frequency by constructing a metasurface embedded with cMQWs. Here, the local electric fields are enhanced due to two plasmonic resonances at both the double transition frequency Ω (920 nm) and the SHG frequency 2Ω (460 nm). The absorption of photons at the fundamental frequency Ω in cMQWs will be enhanced by the first plasmonic resonance, while the radiative decay of the cMQWs at the SHG frequency 2Ω will be boosted by the other plasmonic resonance, leading to an efficient SHG. Such metasurfaces also give rise to efficient conversion of the impinging transverse electric field polarization into the desired *z*-direction^17^, enabling normal excitation of the incident beam to expand the usability of the extremely high nonlinear cMQWs.

Figure 3a depicts the designed metasurface structure, which is composed of (from top to bottom) an array of monocrystalline silver nanocubes (110-nm edge length, Fig. 3b), 4 units of cMQWs, a single layer of Al_2_O_3_ (380 nm thick), and a 50-nm TiN layer. The array of monocrystalline silver nanocubes was self-assembled and then transferred on top of the epitaxial TiN/Al_2_O_3_ multilayer (see details in Supplementary Section S1), and an SEM image is shown in Fig. 3b. These homogenously distributed high-quality monocrystalline silver nanocubes (see the HRTEM images in the insets of Fig. 3b) are used to enhance the light field by plasmonic resonance^22,23^. Here, the light field distributions were calculated using the finite element method (FEM), and the results are summarized in Fig. 3c, d. As can be seen, the electric field enhancement at the longer incident wavelength (920 nm) results from the metal–insulator–metal (MIM) plasmonic waveguide-like optical confinement (Fig. 3d), while the shorter SHG wavelength (460 nm) electric field enhancement arises from the action of localized surface plasmon supported by the monocrystalline silver nanocube array (Fig. 3c).

**Fig. 3:**
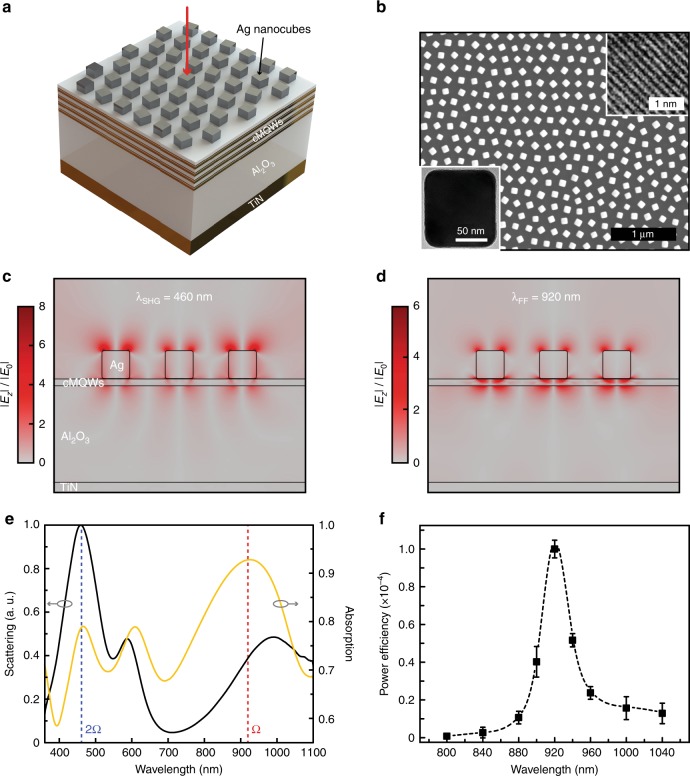
Efficient visible-frequency SHG from cMQWs-embedded monocrystalline silver nanocube metasurface. **a** Schematic of the high SHG efficiency metasurface excited by a normal incidence pump light (thick red arrow). **b** SEM image of the top silver nanocube array. The bottom left inset is a TEM image of a single silver nanocube, while the top right inset shows its HRTEM image where its crystalline lattice can be clearly seen. **c**, **d** Simulated electrical field enhancement (|*E*_*z*_|/|*E*_0_|) for the SHG wavelength (**c**) and the corresponding fundamental wavelength (**d**), respectively. Here, |*E*_0_| is the amplitude of the incident electrical field. **e** Measured scattering (black line) and absorption (yellow line) spectra. Two vertical dashed lines indicate the SHG wavelength (2Ω) and the corresponding fundamental wavelength (Ω), respectively. **f** Wavelength dependence of the power efficiency at the incident pulse intensity of 10 GW/cm^2^. Solid black squares are the experimental result, while the black dashed line is the spline-fitted curve. The visible-frequency SHG power efficiency reaches 10^−4^

Figure 3e shows the measured scattering and absorption spectra. The resonant peak centered at Ω in the absorption spectrum indicates the desired fundamental-frequency absorption enhancement, while the dominant resonant peak centered at 2Ω in the scattering spectrum reveals a high out-coupling efficiency at the SHG wavelength. It is thus expected that there would be a massive enhancement of the visible-frequency SHG efficiency. Here, it is worth noting that the slightly misaligned absorption and scattering peaks at longer wavelengths are due to the fact that the absorption mainly comes from the MIM plasmonic mode, while the scattering involves coupling between the MIM and Ag-cube plasmonic modes supported by the metasurface. The emission spectrum of the cMQWs-embedded metasurface, as excited by a light beam of normal incidence (Fig. 3a), shows a well-behaved visible-frequency SHG. Figure 3f gives the corresponding power efficiency *P*_SHG_/*P*_FF_, where *P*_SHG_ (*P*_FF_) is the average power at the SHG (fundamental) wavelength; it reaches 10^−4^, which is more than four orders of magnitude higher than that of the previous demonstrations of plasmonically enhanced SHG at a visible/NIR wavelength^24–28^. Such a giant visible-frequency SHG power efficiency enables on-chip integration of these optical metasurfaces in ultracompact nonlinear devices.

We have shown a new cMQW material platform that exhibits an extremely high *χ*^(2)^ at the NIR frequency and demonstrated a giant visible-frequency SHG power efficiency in such a cMQW-embedded metasurface. Our material system can be readily integrated into more advanced configurations. For example, by tailoring the quantum states of the cMQWs, high *χ*^(2)^ could be realized in a desired wavelength range; therefore, a broadband efficient SHG is possible by stacking cMQW ultrathin films with different double transition frequencies. Likewise, combinations of optical nonlinearities, e.g., both a high *χ*^(2)^ and a large *χ*^(3)^^9^, would be conceivable in a single material system in a concise manner. Furthermore, a nearly isotropic high *χ*^(2)^ may be achievable in material systems by combining cMQWs with high out-of-plane *χ*^(2)^ and the emerging two-dimensional materials with large in-plane *χ*^(2)^^29^. Nanostructured materials with such giant SHG power efficiencies are expected to enable new on-chip surface nonlinear optical applications.

## Supplementary information

Supplementary information
