# Supplementary material for: Large optical nonlinearity enabled by coupled metallic quantum wells
Source: Light Sci Appl. 2019 Jan 23;8:13. doi: 10.1038/s41377-019-0123-4 (PMC6344563; doi:10.1038/s41377-019-0123-4)
Supplement: Supplementary file 1 — Supplementary information [file 41377_2019_123_MOESM1_ESM.docx]

**Large optical nonlinearity enabled by coupled metallic quantum wells**

Haoliang Qian, Shilong Li, Ching-Fu Chen, Su-Wen Hsu, Steven Edward Bopp, Qian Ma, Andrea R. Tao and Zhaowei Liu

**Contents**

S1. Fabrication of coupled metallic quantum wells metasurface

S2. Visible SHG from coupled metallic quantum wells

S3. Measurement of χ^2^

**S1. Fabrication of coupled metallic quantum wells metasurface**

First of all, coupled metallic quantum wells (cMQWs) composed of TiN and Al_2_O_3_ were epitaxially grown by the reactive magnetron sputtering technique (AJA International)^1^. The grown epitaxial films were characterized by the TEM/HRTEM (see Fig. 1).

Subsequently, silver nanocubes (AgNCs) were synthesized via a polyol synthesis as described in the previous works^2, 3^, where the size of these AgNCs can be well controlled by the concentration of Ag^+^ ion precursor adding in the polyol synthesis process. Here, AgNCs with 110 nm in length were used to form a hexagonal array on air-water interface. The AgNCs colloidal solution chloroform was added dropwise to the air-water interface of the petri dish. The film was allowed to equilibrate about 4-5 hours for hexagonal array formation. The spacing between AgNCs in the hexagonal array can be tuned by the total amount of AgNCs added on the air-water interface.

Finally, the hexagonal array of AgNCs was transferred onto the cMQWs substrates by dip-coating, leading to the plasmonically enhanced nonlinear metasurface.

**S2. Visible SHG from coupled metallic quantum wells**

Figure S1 shows an image of free space setup to observe the visible-frequency SHG emissions from one cMQWs sample. Here, the incident laser pulse was focused by a lens onto the sample that was rotated 45° with respect to the incident beam. The power efficiency reaches 10^-4^ when the incident power is 1000 mW (160 GW/cm^2^ correspondingly). It is so high so that distinct SHG signals along both the transmitted and reflected directions are clearly visible.


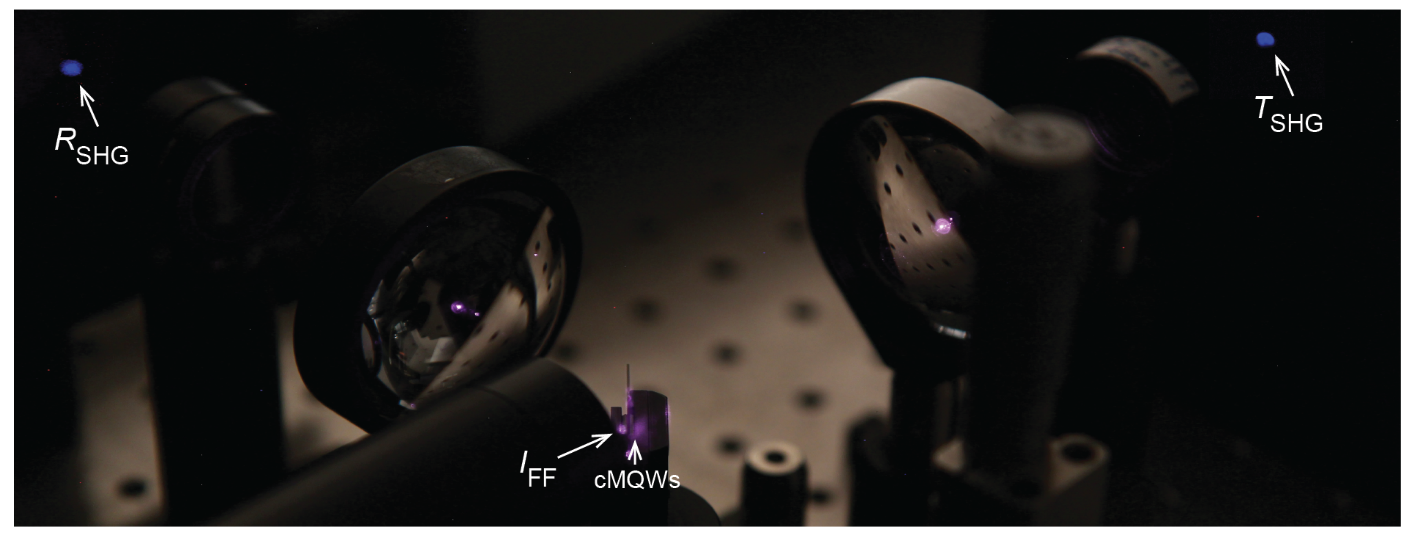


**Figure S1** Image of free space setup where visible SHG from cMQWs is clearly seen.

**S3. Measurement of χ^2^**

The second-order susceptibility (χ^2^) of a single unit of cMQWs was measured according to the following Eq. S1.

$\chi^{(2)}=2\sqrt{\frac{I_{2\omega}}{I_{\omega}^{2}}\frac{n_{2\omega}n_{\omega}^{2}c^{3}\varepsilon_{0}}{2\omega^{2}l^{2}}}$ (S1)

Here, $I_{2\omega}$ is the SHG peak intensity, $I_{\omega}$ is the excitation pulse peak intensity, and, $n_{2\omega}$ and $n_{\omega}$ are the refractive indices of the nonlinear medium at the double frequency and the fundamental frequency, respectively. In addition, $c$ is the speed of light in vacuum, $\varepsilon_{0}$ is the vacuum permittivity, $\omega$ is the fundamental angular frequency, and, $l$ is the interaction length of the nonlinear medium.

The refractive index of the cMQWs sample was extracted from the reflection/transmission measurement as previously reported^4^, and the results from the single unit cMQW sample are shown in Fig. S2.

**
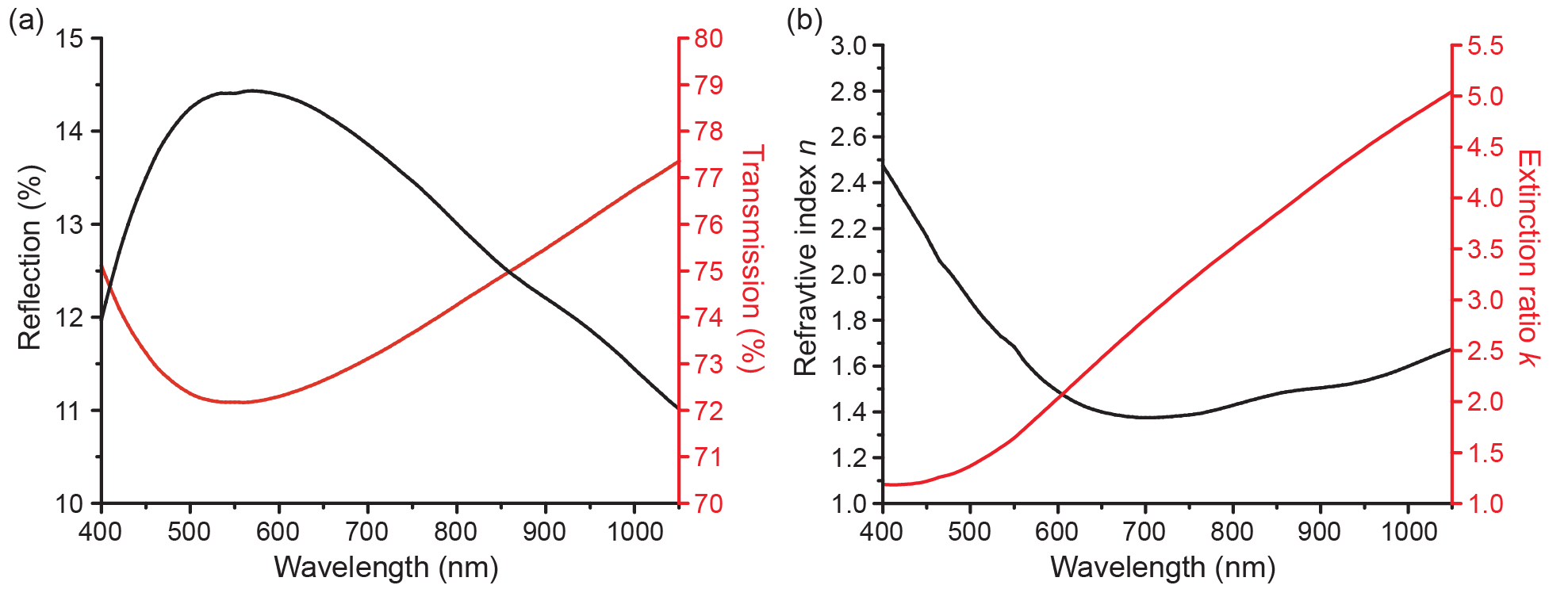
**

**Figure S2** (**a**) Reflection and transmission of the single unit of cMQWs with 30° and *p*-polarization incident beam. (**b**) Refractive index *n* and extinction ratio *k* of TiN in the single unit of cMQWs.

**References**

1. Patsalas P, Kalfagiannis N, Kassavetis S. Optical Properties and Plasmonic Performance of Titanium Nitride. *Materials* 2015; **8**: 3128-3154.

2. Tao A, Sinsermsuksakul P, Yang P. Polyhedral Silver Nanocrystals with Distinct Scattering Signatures. *Angew Chem Int Ed* 2006; **45**: 4597-4601.

3. Sun Y, Xia Y. Shape-Controlled Synthesis of Gold and Silver Nanoparticles. *Science* 2002; **298**: 2176-2179.

4. Qian H, Xiao Y, Liu Z. Giant Kerr response of ultrathin gold films from quantum size effect. *Nat Commun* 2016; **7**: 13153.
